# Hypertensive patients’ knowledge of cardiovascular disease in Calabar, Nigeria

**DOI:** 10.1186/s42506-020-00045-y

**Published:** 2020-07-09

**Authors:** Ogban Omoronyia, Agam Ayuk, Kenneth Nwafor, Annette Legogie

**Affiliations:** 1 Department of Community Medicine, University of Calabar, Calabar, Nigeria; 2 Department of Family Medicine, University of Calabar, Calabar, Nigeria

**Keywords:** Cardiovascular disease, Hypertension, Knowledge, Prevention, Calabar, Nigeria

## Abstract

**Background:**

This study aimed at assessing knowledge of cardiovascular disease risk factors and prevention, among hypertensive patients in a developing country setting with high cardiometabolic disease morbidity and mortality.

**Methods:**

Using descriptive cross-sectional study design and random sampling method, a 30-item questionnaire was used to obtain quantitative data on knowledge of key aspects of cardiovascular disease and practice of preventive measures among hypertensive patients in Calabar.

**Result:**

Two hundred and twelve (212) respondents were surveyed with mean age of 45.5 ± 10.8 years. Approximately two-thirds of respondents (67.9%) had unsatisfactory level of knowledge of CVD. Most respondents had unsatisfactory level of knowledge of appropriate diet (77.4%), basic epidemiology (80.2%), risk factors (63.2%), and symptoms of CVD (74.5%). The mean score for knowledge of risk factors was significantly higher among females compared with males (*p* < 0.05). Consumers compared with non-consumers of alcohol had significantly lower mean scores for knowledge of appropriate diet and symptoms of CVD (*p* < 0.05). Except for medical knowledge where mean score of knowledge was significantly higher among respondents occasionally exercised or consumed fresh fruits compared with other groups, there was no significant difference in mean score of knowledge for other components comparing the various categories of active exercise and consumption of fresh fruits (*p* > 0.05).

**Conclusion:**

Unsatisfactory level of knowledge of cardiovascular disease among hypertensive patients in the study setting was high. There is urgent need to improve efforts and strategies for health education and counseling of patients toward sustainable adoption of preventive behaviors.

## Introduction

In both developed and developing countries, cardiovascular diseases (CVDs) including coronary heart disease, heart failure, and stroke, are a leading cause of adult morbidity and mortality in men and women [1]. In developing countries, the socio-economic impact and catastrophic health expenditure attributable to the rising burden of CVD may be contributing to a worsening state of poverty in many settings [2]. Fortunately, CVDs are largely preventable, with potential minimization of such negative impact, if knowledge-based practice of preventive measures are initiated and sustained especially by high-risk individuals [3]. Hypertensive patients are one of such group of individuals at higher risk of CVD events compared with the general population. Their regular practice of preventive measures will contribute significantly to reduction of morbidity and mortality attributable to CVDs [3]. Yet, the practice of these measures is largely dependent on level of knowledge of CVD and its prevention. Having basic knowledge of the causes/risk factors, clinical manifestations, and prevention of CVDs, is key to sustaining prevention practices [4].

In-patient admission at medical wards and out-patient clinic consultations, affords one of the best opportunities for interaction and provision of individual and group cardiovascular health education to hypertensive and other at-risk patients [5]. Such interaction with nurses, doctors, and other relevant health care workers, is expected to improve knowledge of CVDs and support through cost-effective and practical means of sustainable practice of preventive measures [5]. Unfortunately, in many health facilities in typical developing country settings, the logistics and other challenges with care provision, limit effective delivery health education [6]. The potential impact of this facility-based limitation, on knowledge and practice of CVD prevention, needs to be assessed as a first step, toward improvement in best cardiovascular health education practices in resource-poor settings [5, 6].

Assessment of level and determinants of knowledge of CVD among hypertension patients and other at-risk individuals is therefore a key step toward effective design and implementation of evidence-based health educational and other intervention measures. Unfortunately, in the Niger-delta region of Nigeria, which has one of the highest burden of cardiometabolic diseases, there is paucity of literature on knowledge of CVD among hypertensive patients [7]. This study aimed at contributing to bridging such knowledge-gap, by assessing factors associated with level of knowledge among hypertensive patients seen in a tertiary health facility in Calabar, Nigeria.

## Subjects and methods

### Study design and sample

Cross-sectional study design was used, and data was obtained from hypertensive patients seen at the University of Calabar Teaching Hospital (UCTH), Calabar, within 14 weeks beginning May 2019. Sample size of 206 was calculated using Leslie Kish formula for single proportion [8], *N* = *Z*^2^ *pq*/*d*^2^, where *N* is the required sample size, *Z* is the coefficient at 95% confidence interval (1.96), *p* is the population proportion (being 0.14 from the previous study) [9], *q* is equal to 1 − *p*, and *d* is the acceptable error (0.05). Calculated minimum sample size was 185, which was increased to 206 considering 10% non-response.

During the study period, medical wards (*n* = 48) and outpatient clinic (*n* = 834) registers of hypertensive patients were used as sampling frames, respectively. Systematic random sampling method (with estimated sampling interval of 4) was used to recruit 14 and 194 consenting subjects from the ward and clinic, respectively.

### Data collection

Ethical approval was obtained from UCTH research ethics committee before commencement of the study. A structured questionnaire was used to obtain quantitative data via interviewer-administration. Section one of the questionnaire consisted of sociodemographic and behavioral characteristics including alcohol consumption, smoking, physical activity, and consumption of fresh fruits. Section two consisted of a pretested 28-item set of questions, which had previously been validated [10], but further content validity was done by experts to ensure clarity of questions and focus on study objectives. This section assessed five [5] subheadings including appropriate diet, medical knowledge, basic epidemiology, risk factors, and symptoms of cardiovascular disease, with options of “true,” “false,” and “I don’t know” for each item. Correct response to each of the 28 items contributed a point to the component as well as total knowledge score. A score of at least 60.0% was considered satisfactory and vice-versa (in tune with Blooms criteria), for each component as well as total knowledge score.

### Statistical analysis

Sociodemographic and behavioral characteristics were assessed and compared with level of knowledge using chi-square, independent *t* test, and ANOVA. Data was entered and analyzed using SPSS version 21.0. *P* value was set at 0.05.

## Results

Two hundred and twelve (212) respondents provided complete data. The mean age was 45.5 ± 10.8 years, and most subjects (61.8%) were males (Table 1) More than half of subjects were within 30 to 59 years old (79.2%), married (84.0%), had tertiary level of education (57.5%), and were recruited from the clinic (91.5%). A little above one-third (35.5%) consumed alcohol, while 25.5% and 10.4% consumed fresh fruits and did active physical exercise three or more times weekly, respectively. Most subjects (98.1%) were non-smokers (Table 2).

**Table 1:** Sociodemographic characteristics of respondents (*N* = 212) Calabar, Nigeria

Variable	Frequency	Percentage
Sex		
Male	131	61.8
Female	81	38.2
Age group (in years)		
< 30	14	6.6
30-39	50	23.6
40-49	71	33.4
50-59	47	22.2
≥ 60	30	14.2
Marital status		
Married	178	84.0
Single	28	13.2
Divorced/separated	2	0.9
Widowed	4	1.9
Educational level		
None	4	1.9
Primary	14	6.6
Secondary	72	34.0
Tertiary	122	57.5
Occupation		
Civil servant	110	51.9
Public servant	34	16.0
Business/trader	60	28.3
Others	8	3.8
Source of respondent		
Ward	18	8.5
Clinic	194	91.5

**Table 2:** Behavioral characteristics of hypertensive respondents (*N* = 212)

Variable	Frequency	Percentage
Consume alcohol		
Yes	76	35.8
No	136	64.2
Smoke		
Yes	4	1.9
No	208	98.1
Active physical exercise^*^		
Never	24	11.3
Occasionally (less than once a week)	72	34.0
Once or twice weekly	94	44.3
Three or more times weekly	22	10.4
Consume fresh fruits**		
Never	18	8.5
Occasionally (less than once a week)	56	26.4
Once or twice weekly	84	39.6
Three or more times weekly	54	25.5

*Sufficient physical exercise defined as 30 min of active or 50 min of passive activity within 24 h

**Sufficient fresh fruit consumption defined as 3 to 5 servings within 24 h

One hundred and thirty (130) respondents (61.3%) knew of the low cholesterol content of most vegetables (Table 3). However, only one-fifth (19.8%) of respondents knew of the relatively unhealthy nature of trans fats, while approximately a quarter each, knew of healthy nature of polyunsaturated fats (23.6%), cholesterol content of egg yolk (25.5%), and role of dietary fiber in lowering cholesterol (27.4%). Nearly three-quarters of respondents (73.6%) were aware of heart diseases as the leading cause of death in Nigeria. However, more than half of respondents did not know which of pre- or post-menopausal women were at greater risk of heart disease (57.5%), which disease had worse prognosis between breast cancer and heart disease (70.8%), and the chronic nature of heart disease (77.4%). One hundred and twenty-eight (128) respondents (60.4%) did not know of the functional role of cardiopulmonary resuscitation (CPR), while half (50.0%) knew of HDL and LDL as good and bad cholesterols, respectively. Assuming that most people could tell whether they had high blood pressure, belief in rapid breathing as the best kind of exercise, and consideration of 110/80 mmHg or higher to be high blood pressure, was reported by 42.5%, 38.7%, and 34.9% of respondents, respectively.

**Table 3:** Distribution of knowledge of cardiovascular disease (*N* = 212)

S/n	Variable/question	Correct response	Incorrect response^
		*N* (%)	*N* (%)
Dietary knowledge			
1	Polyunsaturated fats are healthier for the heart than saturated fats.	50 (23.6)	162 (76.4)
2	Trans-fats are healthier for the heart than most other kinds of fats.	42 (19.8)	170 (80.2)
3	Most of the cholesterol in an egg is in the white part of the egg.	54 (25.5)	158 (74.5)
4	Dietary fiber lowers blood cholesterol.	58 (27.4)	154 (72.6)
6	Many vegetables are high in cholesterol.	130 (61.3)	82 (38.7)
Knowledge of basic meaning and epidemiology			
7	Women are less likely to get heart disease after menopause than before.	90 (42.5)	122 (57.5)
8	Heart disease is the leading cause of death in Nigeria.	156 (73.6)	56 (26.4)
9	Most women are more likely to die from breast cancer than heart disease.	62 (29.2)	150 (70.8)
10	Heart disease is better defined as a short-term illness than a chronic illness.	48 (22.6)	164 (77.4)
Medical knowledge			
11	Most people can tell whether or not they have high blood pressure.	122 (57.5)	90 (42.5)
12	The best kind of exercise involves rapid breathing for a sustained period of time.	130 (61.3)	82 (38.7)
13	A healthy person’s pulse should return to normal within 15 min after exercise.	132 (62.3)	80 (37.7)
14	Cardiopulmonary resuscitation (CPR) helps to clear clogged blood vessels.	84 (39.6)	128 (60.4)
15	HDL refers to “good” cholesterol, and LDL refers to “bad” cholesterol.	106 (50.0)	106 (50.0)
17	“High” blood pressure is defined as 110/80 (systolic/diastolic) or higher.	138 (65.1)	74 (34.9)
Knowledge of risk factors			
18	Having had chicken pox increases the risk of getting heart disease.	120 (56.6)	92 (43.4)
19	Eating a lot of red meat increases heart disease risk.	132 (62.3)	80 (37.7)
20	The most important cause of heart attacks is stress.	84 (39.6)	128 (60.4)
21	Walking and gardening are types of exercise that can lower heart disease risk	160 (75.5)	52 (24.5)
22	Smokers are more likely to die of lung cancer than heart disease.	40 (18.9)	172 (81.1)
23	Taking an aspirin each day is thought to decrease the risk of getting heart disease.	58 (27.4)	154 (72.6)
24	Taller people are more at risk for getting heart disease.	104 (49.1)	108 (50.9)
25	People who have diabetes are at higher risk of getting heart disease.	100 (47.2)	112 (52.8)
26	Eating a high fiber diet increases the risk of getting heart disease.	72 (34.0)	140 (66.0)
Knowledge of symptoms			
27	Turning pale or gray is a common symptom of having heart attack.	60 (28.3)	152 (71.7)
28	Sudden trouble seeing in one eye is a common symptom of having a heart attack.	82 (38.7)	130 (61.3)
29	Feeling weak, lightheaded, or faint is a common symptom of having a heart attack.	72 (34.0)	140 (66.0)
30	Men and women experience many of the same symptoms of a heart attack.	58 (27.4)	154 (72.6)

^: including both incorrect and do not know responses

One hundred and twenty (120) respondents knew that prior history of chicken pox (56.6%) and eating a lot of red meat (62.3%) increased the risk of heart disease. Approximately a quarter, half, and two-thirds did not know the heart disease risk-lowering effect of walking or gardening (24.5%), considered taller people to be at higher risk of heart disease (50.9%), and considered high fiber diet to increase risk of heart disease (66.0%), respectively. Most respondents did not know of stress as key cause of heart attack (60.4%), heart disease as the likely cause of death among smokers (81.1%), and a higher risk of heart disease among diabetics (52.8%). Most respondents did not know that men and women experienced many of the same symptoms of heart attack (72.6%), and that fatigue and fainting were symptoms of heart attack (66.0%). However, most respondents considered turning pale or gray (71.7%) and sudden unilateral loss of vision (61.3%) as symptoms of heart attack (Table 3).

Approximately one-third of respondents (32.1%) had satisfactory knowledge of CVD. A little above half of respondents (51.4%) had satisfactory medical knowledge of cardiovascular diseases. However, most respondents had unsatisfactory knowledge of appropriate diet (79.7%), basic epidemiology (81.6%), risk factors (64.6%), and symptoms of CVD (75.5%) (Table 4).

The mean score for knowledge of risk factors was significantly higher among females compared with males (*p* < 0.05, Table 5). The mean score for knowledge of appropriate diet was significantly higher among respondents that were 60 years or older compared with those who were younger, as well as among those who had at least secondary compared with primary or lower level of education (*p* < 0.05). Compared with other occupational groups, public servants had a significantly higher mean score for knowledge of appropriate diet and basic epidemiology, while civil servants had higher mean score for knowledge of risk factors of CVD (*p* < 0.05).

**Table 4:** Distribution of sub-scores for knowledge of CVD (*N* = 212)

Variable	Frequency	Percentage
Dietary knowledge		
Unsatisfactory (< 3)	169	79.7
Satisfactory (≥ 3)	37	20.3
Total	212	100
Mean ± SD (range)	1.58 ± 1.1 (0-4)	
Basic Epidemiology		
Unsatisfactory (< 2)	173	81.6
Satisfactory (≥ 2)	39	18.4
Total	212	100
Mean ± SD (range)	1.68 ± 1.0 (0-4)	
Medical knowledge		
Unsatisfactory (< 3)	103	48.6
Satisfactory (≥ 3)	109	51.4
Total	212	100
Mean ± SD (range)	3.36 ± 1.5 (0-6)	
Knowledge of Risk factors		
Unsatisfactory (< 5)	137	64.6
Satisfactory (≥ 5)	75	35.4
Total	212	100
Mean ± SD (range)	4.10 ± 1.7 (0-8)	
Knowledge of Symptoms		
Unsatisfactory (< 2)	160	75.5
Satisfactory (≥ 2)	52	24.5
Total	212	100
Mean ± SD (range)	1.28 ± 1.3 (0-4)	
Total knowledge score %		
Unsatisfactory (< 60.0%)	144	67.9
Satisfactory (≥ 60.0%)	68	32.1
Total	212	100
Mean % ± SD (range)	42.85 ± 11.4 (14.3-67.9)

**Table 5:** Sociodemographic factors associated with knowledge of CVD (*N* = 212)

Variable	Total score	Dietary	Epid.	Medical	Risk	Symptoms
	Mean % (SD)	Mean (SD)	Mean (SD)	Mean (SD)	Mean (SD)	Mean (SD)
Sex						
Male	41.4 ± 10.4	1.66 ± 1.1	1.58 ± 0.9	3.25 ± 1.6	3.85 ± 1.7	1.25 ± 1.3
Female	45.2 ± 12.5	1.43 ± 1.1	1.84 ± 1.0	3.53 ± 1.4	4.51 ± 1.7	1.33 ± 1.2
*t* test (*p* value)	2.33 (0.02)	1.5 (0.12)	1.9 (0.06)	1.3 (0.19)	2.7 (0.01)	0.5 (0.65)
Age group (in years)						
< 30	45.4 ± 9.30	1.86 ± 0.9	1.79 ± 0.6	3.50 ± 1.3	4.57 ± 1.7	1.00 ± 1.0
30-39	42.8 ± 12.7	1.50 ± 1.1	1.74 ± 1.0	3.46 ± 1.4	4.02 ± 2.1	1.26 ± 1.4
40-49	42.5 ± 11.2	1.48 ± 1.1	1.79 ± 0.9	3.20 ± 1.5	4.18 ± 1.7	1.24 ± 1.3
50-59	44.4 ± 11.7	1.38 ± 1.1	1.53 ± 0.9	3.64 ± 1.4	4.34 ± 1.5	1.53 ± 1.3
≥ 60	40.4 ± 11.4	2.10 ± 1.0	1.50 ± 1.1	3.07 ± 1.8	3.47 ± 1.3	1.17 ± 1.0
*F* test (*p* value)	0.8 (0.55)	2.7 (0.00)	0.9 (0.49)	1.0 (0.42)	1.6 (0.17)	0.7 (0.57)
Marital status						
Married	42.9 ± 12.1	1.62 ± 1.1	1.71 ± 1.0	3.26 ± 1.6	4.09 ± 1.7	1.35 ± 1.3
Unmarried	42.4 ± 5.68	1.35 ± 0.9	1.53 ± 0.5	3.88 ± 1.1	4.18 ± 1.9	0.94 ± 1.2
*t* test (*p* value)	0.2 (0.81)	1.3 (0.19)	1.0 (0.32)	2.2 (0.03)	0.3 (0.79)	1.7 (0.08)
Educational level						
Primary or none	40.9 ± 43.0	1.53 ± 1.0	1.89 ± 1.2	3.33 ± 1.0	3.33 ± 1.9	0.78 ± 0.9
At least secondary	43.0 ± 11.1	2.11 ± 1.2	1.66 ± 0.9	3.36 ± 1.5	4.18 ± 1.7	1.33 ± 1.3
*t* test (*p* value)	0.8 (0.44)	2.3 (0.03)	1.0 (0.33)	0.1 (0.94)	2.0 (0.05)	1.8 (0.07)
Occupation						
Civil servant	42.8 ± 11.3	1.09 ± 0.9	1.65 ± 0.8	3.51 ± 1.2	4.47 ± 1.9	1.25 ± 1.2
Public servant	45.8 ± 9.41	2.18 ± 1.1	1.94 ± 1.1	3.41 ± 2.0	3.76 ± 1.2	1.53 ± 1.4
Business/trader	42.4 ± 11.4	2.10 ± 0.8	1.70 ± 1.0	3.07 ± 1.7	3.70 ± 1.4	1.30 ± 1.1
Others	34.8 ± 16.1	1.75 ± 1.2	0.75 ± 1.4	3.25 ± 1.2	3.50 ± 1.9	0.50 ± 0.5
*F* test (*p* value)	2.2 (0.09)	20.6 (0.00)	3.5 (0.02)	1.2 (0.33)	3.8 (0.01)	1.5 (0.21)
Source of respondent						
Ward	41.3 ± 6.5	2.11 ± 0.8	2.00 ± 0.7	3.22 ± 1.1	3.33 ± 1.0	0.89 ± 0.3
Clinic	43.0 ± 11.7	1.53 ± 1.1	1.65 ± 1.0	3.37 ± 1.5	4.18 ± 1.7	1.32 ± 1.3
*t* test (*p* value)	0.6 (0.53)	2.3 (0.03)	1.5 (0.14)	0.4 (0.69)	2.0 (0.05)	1.4 (0.16)

The mean score of knowledge of appropriate diet was significantly higher among respondents who were in the wards compared with those in the clinic (*p* < 0.05) (Table 5).

Consumers compared with non-consumers of alcohol had significantly lower mean scores for knowledge of appropriate diet and symptoms of CVD (*p* < 0.05) (Table 6). Smokers compared with non-smokers had significantly higher mean score for knowledge of appropriate diet and basic epidemiology of CVD (*p* < 0.05). Except for medical knowledge where the mean score of knowledge was significantly higher among respondents that occasionally exercised or consumed fresh fruits compared with other groups, there was no significant difference in the mean score of knowledge for other components comparing the various categories of active exercise and consumption of fresh fruits (*p* > 0.05).

**Table 6:** Behavioral risk factors associated with knowledge of CVD (*N* = 212)

Variable	Total score	Dietary	Epid.	Medical	Risk	Symptoms
	Mean% (SD)	Mean (SD)	Mean (SD)	Mean (SD)	Mean (SD)	Mean (SD)
Consume alcohol						
Yes	41.6 ± 11.8	1.24 ± 1.0	1.76 ± 0.9	3.37 ± 1.4	4.26 ± 1.9	1.03 ± 1.3
No	43.5 ± 11.1	1.76 ± 1.0	1.63 ± 1.0	3.35 ± 1.5	4.01 ± 1.6	1.43 ± 1.2
*t* test (*p* value)	1.2 (0.24)	3.6 (0.00)	1.0 (0.34)	0.1 (0.94)	1.0 (0.31)	2.3 (0.03)
Smoke						
Yes	57.1 ± 12.4	3.0 ± 1.2	3.0 ± 1.2	4.0 ± 0.0	5.0 ± 1.2	1.0 ± 0.0
No	42.6 ± 11.2	1.6 ± 1.0	1.7 ± 0.9	3.4 ± 1.5	4.1 ± 1.7	1.3 ± 1.2
*t* test (*p* value)	2.6 (0.01)	2.7 (0.01)	2.8 (0.01)	0.9 (0.39)	1.1 (0.29)	0.5 (0.65)
Active exercise						
Never	39.9 ± 12.3	1.58 ± 1.1	1.75 ± 0.9	2.17 ± 1.2	4.25 ± 1.7	1.42 ± 1.1
Occasionally (<once/week)	44.8 ± 12.3	1.42 ± 1.1	1.72 ± 1.0	3.86 ± 1.4	4.25 ± 1.9	1.31 ± 1.3
Once or twice weekly	42.4 ± 10.4	1.68 ± 1.0	1.68 ± 0.9	3.26 ± 1.5	4.00 ± 1.4	1.26 ± 1.2
≥ 3 times weekly	41.6 ± 10.4	1.64 ± 1.2	1.45 ± 1.2	3.45 ± 1.3	3.91 ± 1.5	1.18 ± 1.4
*F* test (*p* value)	1.4 (0.23)	0.9 (0.46)	0.5 (0.69)	8.8 (0.00)	0.4 (0.72)	0.2 (0.92)
Consume fresh fruits						
Never	37.7 ± 8.68	1.22 ± 0.9	1.43 ± 0.9	2.22 ± 0.9	4.11 ± 1.4	1.11 ± 1.1
Occasionally (<once/week)	44.9 ± 11.7	1.71 ± 1.1	1.78 ± 0.9	3.61 ± 1.6	3.96 ± 1.5	1.39 ± 1.3
Once or twice weekly	41.6 ± 10.7	1.64 ± 0.9	1.89 ± 0.6	3.29 ± 1.6	4.00 ± 1.7	1.29 ± 1.3
≥ 3times weekly	44.4 ± 12.2	1.44 ± 1.2	1.89 ± 1.1	3.59 ± 1.3	4.41 ± 2.0	1.22 ± 1.1
*F* test (*p* value)	2.6 (0.05)	1.4 (0.25)	3.4 (0.02)	4.7 (0.00)	0.8 (0.50)	0.3 (0.83)

The mean total knowledge score was significantly higher among females compared with males (*p* < 0.05, Table 5). Compared with other occupations, public servants had a higher total knowledge score, though this difference was not statistically significant (*p* > 0.05). There was no significant difference in total knowledge score comparing marital status, educational level, and source of recruitment (*p* > 0.05).

Compared with non-smokers, smokers had a significantly higher mean total knowledge score (*p* < 0.05). Respondents that consumed fresh fruits at least once weekly had a higher level of mean total knowledge score, though this difference was marginally significant (*p* = 0.05, Table 6). Consumption of alcohol and active physical exercise were not found to be associated with total knowledge score (*p* > 0.05).

## Discussion

This study was aimed at assessing knowledge of CVD among hypertensive patients seen in a developing country setting. There was unsatisfactory knowledge of CVD among subjects in all areas assessed (Table 3 and 4). Similar studies, in South West [11], North West [12], and North Central Nigeria [13], found unsatisfactory knowledge of 52.9%, 53.1%, 58.9% in hypertensive patients about cardiovascular disease, respectively. This suggests possibility of overemphasis on clinical treatment of patients, at the expense of health education during clinical consultations and other forms of contact with healthcare workers in our health facilities. Unsatisfactory level of knowledge may be contributing to non-compliance with pharmacologic and non-pharmacologic management of CVDs [14, 15]. The beneficial effect of diffusion of preventive health information to family and friends is also lost with this scenario [16].

In this study, females compared with males had a higher level of knowledge in virtually all areas of CVD assessed, but with statistical significance only in risk factors for CVD (Table 5). However, it was expected that males would be more aware that their sex constitutes a non-modifiable risk factor for CVD [17]. Females in the study setting may be more inquisitive of health information obtained via diverse sources, including more frequent access to available maternity and other healthcare service compared with males [18, 19]. Also, subjects with higher level of education had better knowledge of virtually all areas of CVD, but with statistical significance for dietary recommendations and risk factors for CVD. This difference may be due to more educational interaction and exposure to diverse formal and informal sources of health information by more educated subjects [20]. Heath literacy which is required for better understanding and application of health information is usually better among the more educated individuals [21, 22]. This underscores the need for improvement in school enrolment and reduction in school drop-out in developing countries where education is mostly sponsored out-of-pocket with little or no contribution from governments [23].

Subjects recruited from the ward compared with clinic, had significantly better knowledge of dietary recommendations for CVD prevention. This may result from more frequent and longer duration of exposure to nutritional health information, especially via the special diet they receive as part of inpatient treatment. High burden of out-patient consultation in public hospitals in developing countries, may be limiting the duration of patient-doctor interaction for better delivery of nutritional and other relevant aspects of cardiovascular health education [24]. This underscores the need for control of the current brain drain of medical personnel in developing countries, toward meeting the unmet need for better patient interaction with health personnel [24]. Also, consumers compared with non-consumers of alcohol had significantly lower level of knowledge of symptoms and appropriate diet for prevention of CVD. This unsatisfactory level of knowledge may be contributing to initiation and/or sustenance of practice of alcohol consumption among hypertensive patients in the study setting. In other words, consumers of alcohol may not have adequate knowledge of recommended dietary practices including threshold levels of alcohol consumption [25]. They may also be unaware of the potentially innocuous onset of symptoms and complications of CVD. This suggests the need for improved cardiovascular health education and counseling, especially among hypertensive patients with history of alcohol consumption [25]. Significantly, higher level of knowledge of epidemiology of CVD was found among more frequent compared with less frequent consumers of fresh fruits. Knowledge of high burden of CVD among adults may be a motivating factor for initiation and maintenance of fruit consumption, despite lack of significant differences in the level of knowledge of dietary recommendations for CVD prevention [26].

### Limitations of the study

There are notable limitations of this study. Respondents from tertiary health facility, may not be representative of the majority of hypertensive patients who access secondary or primary health facilities in the study region. This is due to the potential provision of better individual and group health education and counseling, in tertiary compared with non-tertiary health facilities. In other words, conduction of study among less literate rural or suburban respondents in non-tertiary health facilities may have yielded poorer knowledge of CVD. Also, considering that many hypertensive patients have poor access to available healthcare services that contribute to their knowledge, the true proportion with adequate knowledge of CVD may have been lower if the study was community-based. Caution should therefore be exercised in the interpretation and application of findings of this study.

## Conclusion

There is a high degree of unsatisfactory level of knowledge of cardiovascular diseases among hypertensive patients in the study setting. There is a need for healthcare workers and policymakers to redouble effort in cardiovascular health education toward control/prevention of CMD and attainment of health-related SDGs in developing countries. Such efforts should consider key factors associated with knowledge of CVDs, which may vary in different settings. Trained nurse-led individual and group cardiovascular health education, delivered during out-patient visits prior to medical consultation, may be useful for improvement in knowledge of CVDs to hypertensive patients in developing countries. Further study with inclusion of hypertensive patients in non-tertiary health facilities and less urban developing country settings is recommended.

## Data Availability

Data supporting study findings will be made freely available to any individual by mail request to corresponding author. There is no restriction to data availability.
